# Intravascular Endothelin-1 does not trigger or increase susceptibility to Spreading Depolarizations

**DOI:** 10.1186/s10194-020-01194-3

**Published:** 2020-10-27

**Authors:** Kazutaka Sugimoto, Andreia Morais, Homa Sadeghian, Tao Qin, David Y. Chung, Messoud Ashina, Anders Hougaard, Cenk Ayata

**Affiliations:** 1 Department of Radiology, Massachusetts General Hospital, Harvard Medical School, 149 13th Street, 6408, Charlestown, MA 02129 USA; 2 Department of Neurosurgery, Yamaguchi University School of Medicine, Yamaguchi, Japan; 3 Department of Neurology, Massachusetts General Hospital, Harvard Medical School, Boston, USA; 4 Danish Headache Center, Department of Neurology, Rigshospitalet Glostrup, Glostrup, Denmark

**Keywords:** Endothelin-1, Spreading depolarization, Migraine aura

## Abstract

**Objectives:**

Spreading depolarizations (SD) likely manifest as aura in migraineurs. Triggers are unknown although vascular events have been implicated. Direct carotid puncture has been reported to trigger migraine with aura. The potent vasoconstrictor endothelin-1 (ET-1), which can be released from the endothelium under pathological conditions, may play a role. Here, we tested whether intracarotid ET-1 infusion triggers SD and whether systemic ET-1 infusion increases the susceptibility to SD.

**Methods:**

Carotid infusions were performed in mice (C57BL/6, male) through a catheter placed at the carotid bifurcation via the external carotid artery. Intracarotid ET-1 (1.25 nmol/ml) was infused at various rates (2–16 μl/min) with or without heparin in the catheter and compared with vehicle infusion (PBS with 0.01% acetic acid) or sham-operated mice (*n* = 5). Systemic infusions ET-1 (1 nmol/kg, *n* = 7) or vehicle (*n* = 7) infusions were performed in rats (Sprague-Dawley, male) via the tail vein. Electrical SD threshold and KCl-induced SD frequency were measured after the infusion.

**Results:**

Intracarotid infusion of saline (*n* = 19), vehicle (*n* = 7) or ET-1 (*n* = 12) all triggered SDs at various proportions (21%, 14% and 50%, respectively). These were often associated with severe hypoperfusion prior to SD onset. Heparinizing the infusion catheter completely prevented SD occurrence during the infusions (*n* = 8), implicating microembolization from carotid thrombi as the trigger. Sham-operated mice never developed SD. Systemic infusion of ET-1 did not affect the electrical SD threshold or KCl-induced SD frequency.

**Conclusion:**

Intravascular ET-1 does not trigger or increase susceptibility to SD. Microembolization was the likely trigger for migraine auras in patients during carotid puncture.

## Background

Spreading depolarization (SD), and associated spreading depression, is a slowly propagating wave of pandepolarization involving all cell types in cerebral gray matter, and likely underlies migraine aura [1]. How such an intense depolarization event is triggered in apparently otherwise healthy brains of migraineurs is unknown. Several lines of evidence implicate vascular mechanisms. For example, direct carotid puncture can trigger aura in migraine patients [2, 3]. Cervical artery dissections are more common in migraineurs [4] and can also present with aura [5]. Patients with arteriovenous malformations [6] or cerebral autosomal dominant arteriopathy with subcortical infarcts and leukoencephalopathy (CADASIL) often present with frequent auras [7]. Sclerotherapy for varicose veins [8] and the agitated saline test to detect right-to-left shunts by ultrasound have been reported to trigger migraine with aura [9]. Finally, migraine with aura is associated with an increased risk of stroke [10].

Endothelin-1 (ET-1) is a potent vasoconstrictor released from endothelium under certain pathological conditions [11]. During migraine attacks, plasma ET-1 can be elevated [12, 13]. Genome-wide association studies found a link between migraine and a gene variant that results in higher expression of the ET-1 and increased binding of ET-1 to ET_A_ receptors on vascular smooth muscle cells [14]. Moreover, in experimental animals topical ET-1 application onto the cortex triggers SD [15]. As such, there has been good reason to hypothesize that ET-1 could be a pathophysiological link between vascular injury (e.g. carotid puncture, dissections) and migraine aura attacks.

Here, we tested the hypothesis that endovascular exposure to ET-1 in the cerebral circulation can trigger or predispose to migraine aura. To this end, we examined whether intracarotid ET-1 infusions trigger cortical SD as the physiological surrogate of aura, and whether systemic intravenous ET-1 infusions alter the overall susceptibility to SD.

## Methods

Experiments were approved by the MGH Institutional Animal Care and Use Committee and carried out in accordance with the Guide for Care and Use of Laboratory Animals (NIH Publication No. 85–23, 1996). Study design and reporting followed ARRIVE guidelines.

### Intracarotid ET-1 infusion experiments

**Fig. 1:**
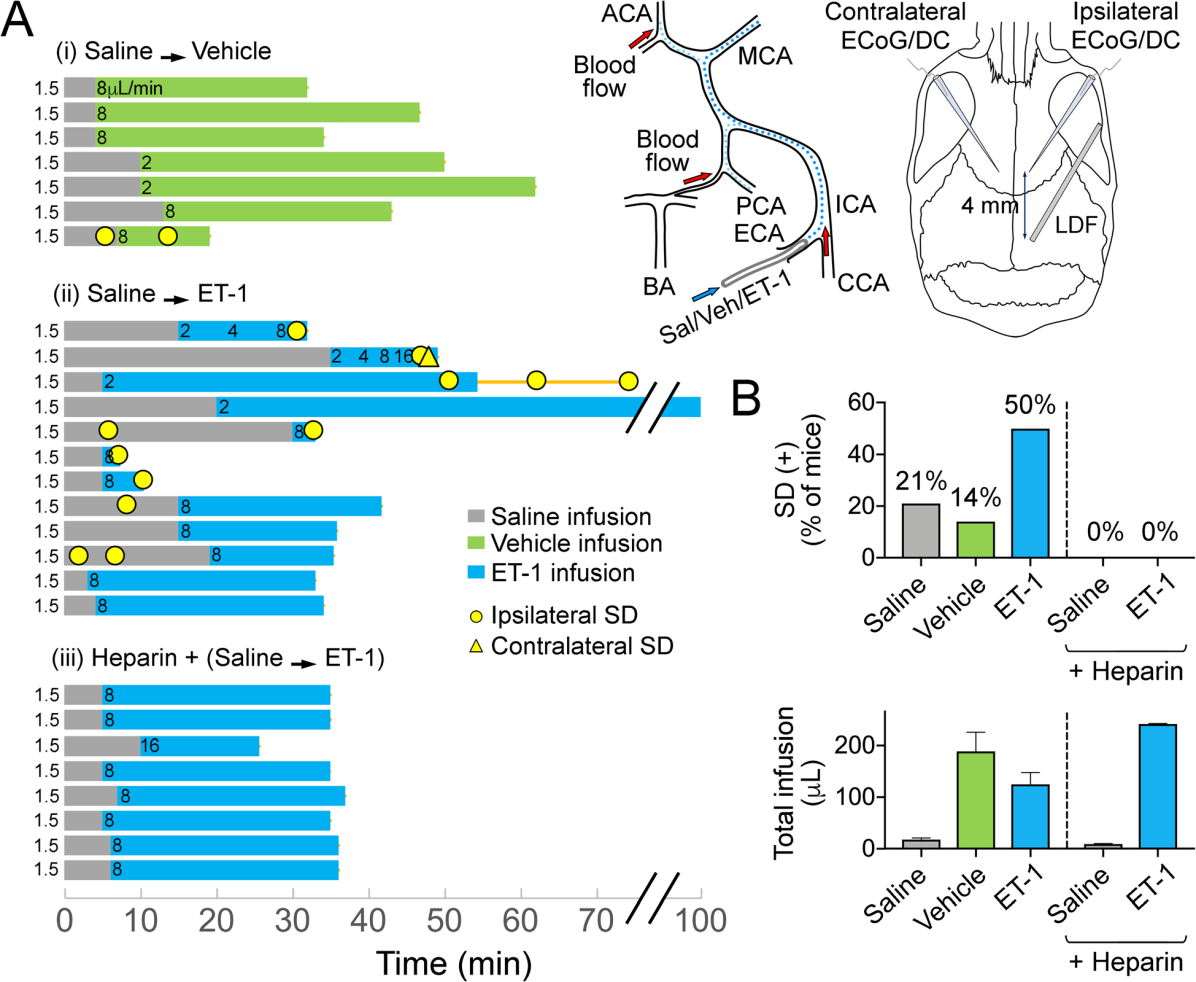
Intraarterial endothelin-1 does not trigger spreading depolarization in mice. **a** Timelines of intracarotid infusion experiments (left) and experimental setup (right). After the surgical preparation, saline was infused from carotid catheter at 1.5 μl/min. Then vehicle or endothelin-1 (ET-1) was infused through the carotid catheter. Numbers on each horizontal bars indicate infusion rate. For carotid infusions, catheter was inserted retrogradely into the external carotid artery and spreading depolarizations (SD) were detected using glass microelectrodes placed on both hemispheres and a laser Doppler on the ipsilateral hemisphere. **b** SD occurrence rates (upper panel) and total infusion volumes (lower panel). Four out of 19 mice (21%) developed SD during saline infusion. During vehicle infusion, one out of 7 mice had SD (14%), while endothelin-1 (ET-1) infusion triggered SD in 6 out of 12 mice (50%, *p* = 0.14, Chi-square test). In the presence of heparin, neither saline nor ET-1 infusion triggered an SD in any of the 8 mice studied (*p* = 0.011 versus no heparin, Chi-square test). Mean ± standard errors are shown

For carotid infusions, 32 mice (C57Bl/6, male, ~ 10 week, ~ 25 g, Charles River Laboratories, Wilmington, MA, USA) were used. Mice were anesthetized with isoflurane (4% induction, 1.5% maintenance in 70% N_2_ and 30% O_2_). A femoral artery catheter was used to monitor arterial blood pressure. Rectal temperature was monitored and maintained at 37 °C via a servo-controlled heating pad. Catheter (Micro-Renathane 0.010″ × 0.005″ per ft. Braintree Scientific, MA, USA) was inserted retrogradely into the external carotid artery and advanced into the carotid bifurcation. This surgical approach preserved common carotid artery flow with the catheter tip at the bifurcation (Fig. 1a). Saline infusion (1.5 μl/min) started immediately to maintain patency. The wound was then closed, and the animal was placed in a stereotaxic frame (David Kopf Instruments, Tujunga, CA). SDs were detected using intracortical glass microelectrodes in both hemispheres (Fig. 1a). For electrophysiological recordings, burr holes were drilled over both hemispheres (1 mm anterior and lateral to the bregma) and covered with mineral oil to prevent drying and consequent SDs. The steady (DC) potential and electrocorticogram (ECoG) were recorded with glass micropipettes filled with 200 mM NaCl and inserted 300 μm below the dural surface (Axoprobe-1A, Axon Instruments, Union City, CA). An Ag/AgCl reference electrode was placed subcutaneously in the neck. In addition, regional cerebral blood flow (CBF) was recorded using laser Doppler flowmetry (LDF; PF2, Perimed, Jarfalla, Sweden). The LDF probe (0.48 mm tip diameter) was placed over the intact skull (3 mm posterior, 1 mm lateral to the bregma) ipsilateral to the infusion. The magnitude of CBF changes were expressed as percent of pre-infusion baseline.

After saline infusion at baseline (1.5 μl/min), vehicle (phosphate-buffered saline with 0.01% acetic acid; *n* = 7) or ET-1 (1.25 nmol/ml; *n* = 12) was infused through the carotid catheter. In a separate cohort, we filled the carotid catheter with heparinized saline prior to ET-1 infusion (*n* = 8). All infusions were continued until an SD was detected or for at least 15 min. Infusion doses and rates are shown in Fig. 1a, chosen based on the literature [16, 17]. We also studied sham-operated mice without infusion (*n* = 5); four of these mice had arterial manipulation but no cannulation and one had an external carotid artery catheter placed without infusion. All syringes and catheters were changed for every experiment to avoid contamination. Because SDs occurred during saline infusion, contributing to our decision to test heparin, these infusion periods were also included in the statistical analyses.

### Intravenous ET-1 infusion experiments

For systemic infusions, we used 14 rats (Sprague-Dawley, male, ~ 275 g, Charles River Laboratories, Wilmington, MA, USA) because of lower failure rates of tail vein injections compared with mice. Rats were anesthetized with isoflurane (4% induction, 1.5% maintenance in 70% N_2_ and 30% O_2_), intubated via a tracheostomy and mechanically ventilated (SAR-830; CWE, Ardmore, PA). A femoral artery catheter was used to monitor arterial blood pressure, pH, pO_2_ and pCO_2_ (Rapidlab 248 blood gas/pH analyzer; Siemens HealthCare, Germany). Rectal temperature was monitored and maintained at 37 °C via a servo-controlled heating pad. Animals were then placed on a stereotactic frame (Stoelting, Wood Dale, IL), and cranial burr holes were drilled under saline cooling overlying occipital (4.5 mm posterior, 2 mm lateral from bregma; 2 mm diameter for stimulation), parietal (1.5 mm posterior, 2 mm lateral from bregma; 1 mm diameter for recording), and frontal (1.5 mm anterior, 2 mm lateral from bregma; 1 mm diameter for recording) cortex (Fig. 3a). Dura was carefully removed, and cortex allowed to rest for 15 min under saline irrigation. The ECoG and DC potential were recorded using glass capillary microelectrodes and a differential amplifier (EX1; Dagan Corporation, Minneapolis, MN), and continuously digitized (PowerLab; ADInstruments, Colorado Springs, CO).

SD susceptibility was measured (Fig. 3a) as previously described [18, 19]. The electrical threshold to induce an SD was determined by direct cortical stimulation using a stimulus isolator (WPI, Sarasota, FL) and a bipolar stimulation electrode (400 μm tip diameter, 1 mm tip separation; FHC, Bowdoin, ME) placed on the pial surface. Single square pulses of stepwise increasing duration and intensity (50–4000 μC) were applied at 5-min intervals until an SD was observed. To determine SD frequency, a cotton ball (1.5–2 mm diameter) soaked with KCl (300 mM) was placed on the occipital cortex and changed every 15 min for 1 h. Only SDs that were 5 mV or larger in amplitude were counted. We did not monitor CBF in these experiments because prior work in mice, cats and dogs has shown no change in CBF upon intravenous ET-1 administrations [16, 17].

We administered ET-1 (1 nmol/kg, *n* = 7) or vehicle (*n* = 7) via the tail vein in a blinded fashion. The dose was chosen based on the literature [20, 21]. The electrical SD threshold and KCl-induced SD frequency were determined in this order in the right hemisphere 5 min after ET-1 infusion. ET-1 infusion was then repeated, and SD susceptibility assessed in the left hemisphere 5 min later as above.

### Statistical analysis

Analyses were performed in a blinded manner. Systemic infusions were randomized. Intracarotid infusions were not randomized due to the dynamic and exploratory nature of this experimental protocol, which also precluded a priori sample size calculations. Data are presented as fraction of total (%), mean ± standard error and whisker-box plots. Statistical analyses were performed using Prism 8 (GraphPad Software, La Jolla, CA), and indicated where the data are presented. SD occurrence in intracarotid infusion experiments was analyzed using Fisher’s least significant difference test. SD threshold and frequency were analyzed using mixed-effects 2-way repeated measures ANOVA (independent variables: infusion and hemisphere) followed by Sidak’s multiple comparisons test. Since hemisphere did not affect the outcome, data were averaged between the hemispheres and presented as such in the figure. Systemic physiological data were analyzed using one-way ANOVA followed by Tukey’s multiple comparisons or t test. *P* < 0.05 was considered statistically significant.

## Results

### Intracarotid ET-1 infusion

Sham groups did not develop SD. A total of 19 mice received saline infusions (1.5 μl/min) prior to vehicle (*n* = 7) or ET-1 (*n* = 12) infusions at various rates and timelines indicated on Fig. 1a. SDs occurred at all stages: 21% of mice developed SD during saline infusion, 14% during vehicle infusion, and 50% during ET-1 infusion (*p* = 0.14, Chi-square test, Fig. 1b upper panel). Although SD occurrence appeared more frequent during ET-1 infusions, when adjusted per volume of infusion it did not differ among the groups (1.22 ± 0.64 SDs/ml saline, 0.14 ± 0.14 SDs/ml vehicle, 1.43 ± 0.53 SDs/ml ET-1; *p* = 0.19, Kruskal-Wallis test; Fig. 1b lower panel).

The occurrence of SDs even during saline or vehicle infusions, and hypoperfusion preceding SDs, implicated carotid emboli as the SD trigger. Therefore, we pretreated the infusion catheter with heparin in a separate cohort (*n* = 8). In the presence of heparin, neither saline nor ET-1 infusion caused CBF drop or triggered an SD in any of the 8 mice studied (*p* = 0.011 versus no heparin, Chi-square test; Fig. 1b upper panel), despite comparable volumes of infusion (Fig. 1b lower panel). These data confirmed that SDs were triggered by embolic focal ischemia.

**Fig. 2:**
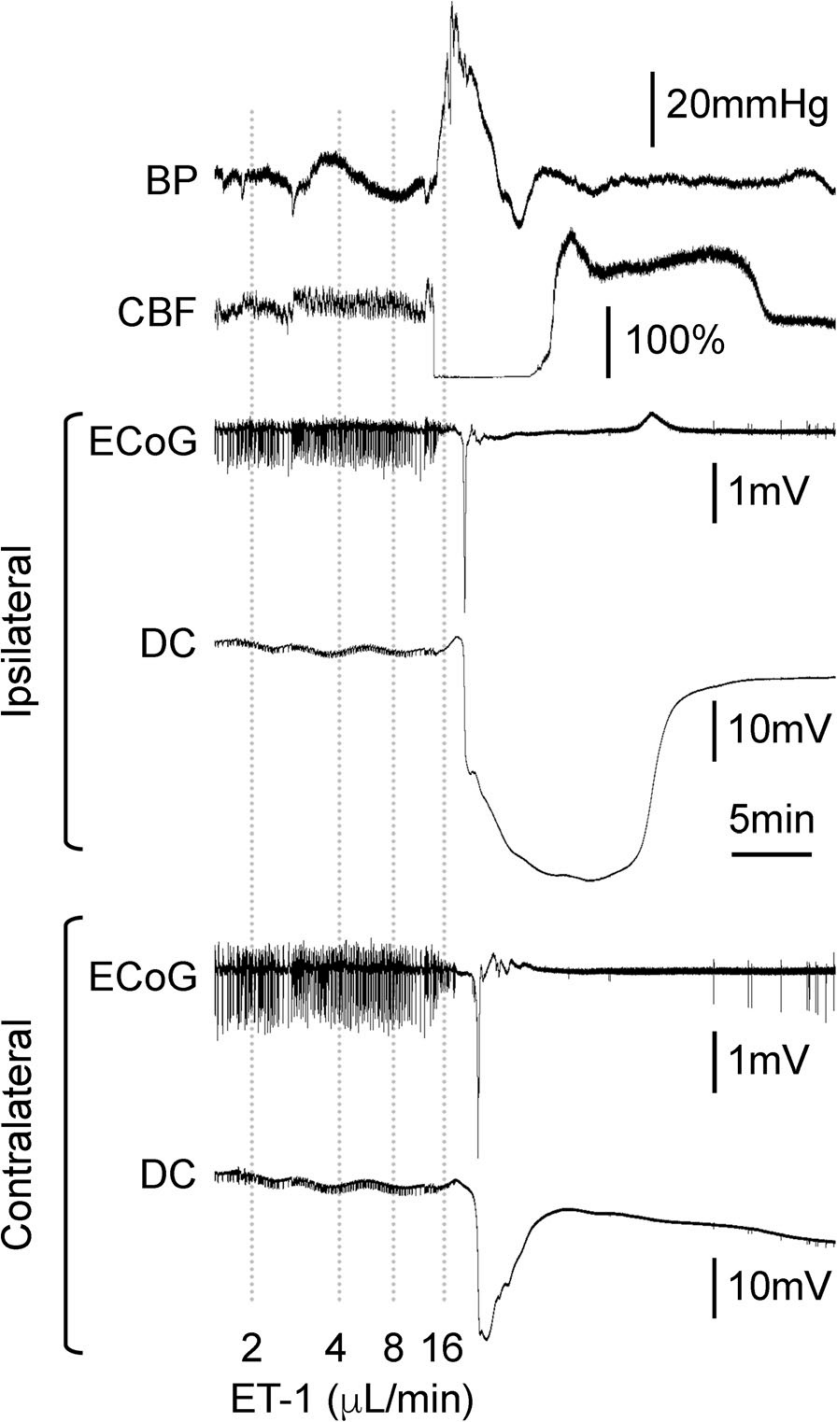
A representative experiment with severe hypoperfusion during endothelin-1 (ET-1) infusion. During an ET-1 infusion rate of 8 μl/min, regional cerebral blood flow (CBF) suddenly dropped to zero and within seconds arterial blood pressure spiked by more than 50 mmHg. This was followed by electrocorticogram (ECoG) suppression and a massive direct coupled (DC) potential shift that resembled anoxic depolarization. Interestingly, contralateral ECoG was also suppressed 1 min after the ipsilateral hemisphere and suffered a depolarization that was shorter lasting

In support of an embolic mechanism, all animals that developed an SD showed severe hypoperfusion (CBF ~ 20% of baseline) preceding the SD, regardless of the infusion solution. An example of SD following severe hypoperfusion during ET-1 infusion is shown in Fig. 2. Shortly after increasing the infusion rate to 8 μl/min, CBF suddenly dropped to zero and within seconds arterial blood pressure spiked by more than 50 mmHg. This was followed by ECoG suppression and a massive DC shift that resembled anoxic depolarization. Interestingly, contralateral ECoG was also suppressed 1 min after the ipsilateral hemisphere and suffered a depolarization that was shorter lasting. The sequence of events was suggestive of a sudden and massive clot embolization into the carotid system, including the anterior cerebral artery, which forms a confluence perfusing the parasagittal cortex in both hemispheres, to explain the contralateral ischemia and SD.

### Intravenous ET-1 infusion

**Fig. 3:**
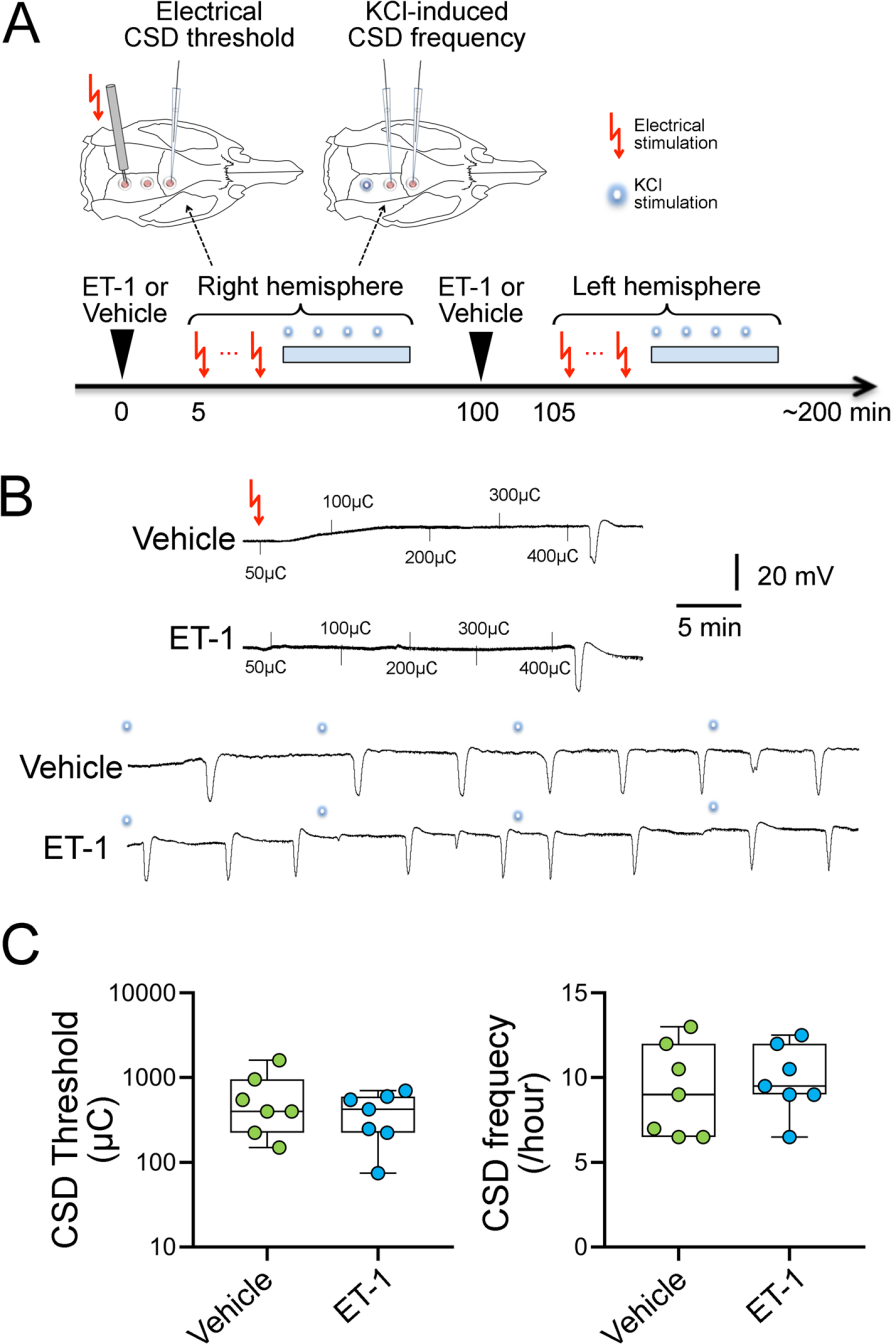
Endothelin-1 does not increase susceptibility to spreading depolarization in rat. **a** At first, endothelin-1 (ET-1) or vehicle was administrated from tail vein in a blinded manner. Single-square pulses of increasing duration and intensity (50–4000 μC) were applied at 5-min intervals until a spreading depolarization (SD) was observed. Then, SD frequency was assessed by topical cortical application of KCl and represented as the number of SDs per hour. After the additional ET-1 or vehicle administration, another hemisphere was studied consecutively. **b** Representative tracings of electrical threshold for SD and SD frequency by topical cortical application of KCl in each group. (C) Whisker box plots (horizontal line, median; box, interquartile range; whiskers, full range) show that endothelin (1 nmol/kg, *n* = 7) did not affect the electrical threshold for SD (vs vehicle, 2way ANOVA with post hoc analysis using Sidak’s multiple comparisons test). Similarly, endothelin also did not alter KCl-induced SD (versus vehicle, 2-way ANOVA followed by Sidak’s multiple comparisons test). The ends of the whiskers represent minimal and maximal data points. The horizontal lines within the box indicate the median. Circles show averaged number of the result of right and left hemispheres

We next tested the effects of systemic ET-1 infusion on SD susceptibility (Fig. 3a). We did not observe any spontaneous SD after intravenous vehicle or ET-1 (1 nmol/kg) infusions. Neither the electrical SD threshold (*p* = 0.33) nor the frequency of KCl-induced recurrent SDs differed between vehicle and ET-1 arms (*p* = 0.39; Fig. 3b,c). Because systemic physiological parameters can affect SD susceptibility [18], we monitored and found them to be within normal limits in both treatment arms (Table 1).

**Table 1:** Systemic physiological parameters

		BP (mmHg)	pH	pCO_2_ (mmHg)	pO_2_ (mmHg)
Intracarotid infusion (mouse)	Vehicle	89 ± 21	7.35 ± 0.07	37 ± 7	152 ± 23
	ET-1	93 ± 16	7.35 ± 0.07	34 ± 8	153 ± 16
	ET-1 + heparin	82 ± 14	7.36 ± 0.04	36 ± 3	171 ± 12
Intravenous infusion (rat)	Vehicle	100 ± 12	7.43 ± 0.02	35 ± 3	133 ± 14
	ET-1	105 ± 11	7.44 ± 0.02	36 ± 4	139 ± 12

*BP* Blood pressure in mmHg. *ET-1* Endothelin-1. Blood gas values are in mmHg. Data are presented as mean ± standard deviation and analyzed using one-way ANOVA followed by Tukey’s multiple comparisons or using t-test. *p* < 0.05 was considered statistically significant. There was no difference between the groups

## Discussion

Our data show that intravascular ET-1 does not trigger or increase susceptibility to SD. These data are congruent with recent reports that intravenous infusion of ET-1 does not induce aura symptoms or headache in healthy volunteers or in patients with a history of migraine with aura [22, 23]. Intraarterial emboli was the likely trigger for SDs observed in our study, given that SDs occurred following sudden and severe cerebral hypoperfusion, regardless of the infusion solution. This was confirmed when pre-heparinization of the carotid catheter abolished both hypoperfusion and SDs. Indeed, microemboli are capable of triggering SD without causing lasting ischemia or injury, at least in experimental animals [24]. Therefore, previous reports of aura in the setting of vascular injury [2, 3, 5] likely reflected embolic events as well. It is important to note, however, that our data do not rule out a role for cerebral endothelial dysfunction or other cerebral vasoconstrictive conditions in triggering or modulating aura attacks.

In contrast to intravascular ET-1, it is well established that application of topical ET-1 (from the adventitial side) causes SD, which is likely due to its potent vasoconstrictive and ischemic effect via the ETA receptor/phospholipase C pathway [15, 17, 25, 26]. In other words, ET-1 has to gain access to the vascular smooth muscle on the abluminal side to cause constriction. It is unlikely that intravascular ET-1, a peptide, crosses the blood brain barrier to any significant extent [27–29]. Indeed, we did not observe a dose-rate-dependent decrease in CBF during ET-1 infusions. Any hypoperfusion we observed was sudden onset and often large magnitude. The latter is in part what prompted us to hypothesize embolic ischemia as a potential mechanism and test heparin. Although SD itself can lead to blood brain barrier disruption after a few hours [30] careful monitoring for accidental SDs during cranial preparation ensured that the blood brain barrier was not disrupted prior to ET-1 infusions in our experiments.

Although SD was not more common in ET-1 group compared with saline when normalized for the volume of infusion, there was a trend for higher proportion of animals exposed to ET-1 to develop severe hypoperfusion followed by SD. Hence, it is still possible that even the lowest infusion rate of ET-1 might have activated platelets and promoted clotting at the catheter tip. However, the effect of ET-1 on platelets has been controversial with conflicting results showing activation in some studies [31, 32], inhibition in others [33, 34] and no effect on human platelets [31, 35, 36].

Our study has limitations. First, heparin may block ET-1 by reducing its expression and release or by interfering with its target action [37]. However, we did not induce ET-1 release but rather directly administered it intravascularly, and we did not infuse heparin into cerebral vasculature along with ET-1 but rather pre-treated the catheters with heparin to prevent local clotting. Nevertheless, at the end of the experiments we bolus injected ET-1 (1 nmol) through the femoral artery catheter, which was always heparinized, and confirmed a hypertensive response (data not shown). Second, intravenous doses of ET-1 may not have achieved sufficient circulating levels due to rapid elimination. We deliberately avoided very high doses in order not to affect the systemic physiology, which is known to alter SD susceptibility. Nevertheless, it is possible that higher intravenous infusion dose-rates might have been efficacious.

## Conclusions

In summary, we show that intravascular ET-1 does not trigger or increase susceptibility to SD. Microembolization was the likely trigger for SDs in our study and migraine auras in patients during carotid puncture. Taken together with recent clinical data, it is unlikely that intravascular ET-1 plays a major causative role as a migraine trigger. Furthermore, our study highlights the importance that future studies using intracarotid infusions must control for embolization and inadvertent triggering of SD.

## Data Availability

The datasets used and/or analysed during the current study are available from the corresponding author on reasonable request.

## Abbreviations

SD: Spreading depolarization
ET-1: Endothelin-1
CADASIL: Cerebral autosomal dominant arteriopathy with subcortical infarcts and leukoencephalopathy
ECoG: Electrocorticogram
CBF: Cerebral blood flow
DC: Direct coupled
LDF: Laser Doppler flowmetry
